# Novel Anthraquinone Derivatives and Their Complexes with Metal Ions with Anticancer Activity: Structure/Redox and Chelation Activity Correlations

**DOI:** 10.3390/ph17121717

**Published:** 2024-12-19

**Authors:** Olga Yu. Selyutina, Maya A. Ul’yanova, Olga A. Chinak, Viktor A. Timoshnikov, Lidiya G. Fedenok, Alexander A. Stepanov, Vadim V. Yanshole, Leonid V. Kulik, Sergey F. Vasilevsky, Nikolay E. Polyakov, George J. Kontoghiorghes

**Affiliations:** 1 Institute of Chemical Kinetics and Combustion, Institutskaya Str. 3, Novosibirsk 630090, Russia; olga.gluschenko@gmail.com (O.Y.S.); m.ulyanova1@g.nsu.ru (M.A.U.); chinakolga@gmail.com (O.A.C.); timoshnikov@kinetics.nsc.ru (V.A.T.); fedenok@kinetics.nsc.ru (L.G.F.); stepanov@kinetics.nsc.ru (A.A.S.); chemphy@kinetics.nsc.ru (L.V.K.); polyakov@kinetics.nsc.ru (N.E.P.); 2 Institute of Chemical Biology and Fundamental Medicine SB RAS, Lavrentyev Ave. 8, Novosibirsk 630090, Russia; 3 International Tomography Center SB RAS, Institutskaya Str. 3a, Novosibirsk 630090, Russia; vadim.yanshole@tomo.nsc.ru; 4 Postgraduate Research Institute of Science, Technology, Environment and Medicine, Limassol CY-3021, Cyprus

**Keywords:** anthraquinones, quinones, chelation, metal complexes, iron complexes, copper complexes, cytotoxic activity, lipid peroxidation, reactive oxygen species, cancer cells A549, NMR

## Abstract

**Background/Objectives:** Some specific anthraquinone derivatives (AQs) are known to be used widely as effective chemotherapeutic agents in the treatment of cancer. However, their fundamental shortcoming is the high rate of cardiotoxicity observed in treated patients, which is thought to be caused by the increase in production of reactive oxygen species (ROS) catalyzed by iron and copper. The development of improved AQs and other anticancer drugs with enhanced efficacy but reduced toxicity remains a high priority. The aim of this study was to evaluate the cytotoxic and ROS production effects of chelate iron and copper complexes of two novel AQs, namely 4-hydroxynaphto[2,3-*h*]cinnoline-7,12-dione (Q2) and 3-(hydroxymethyl)naphto[2,3-*h*]cinnoline-4,7,12(1*H*)-trione (Q3). **Methods**: The chelation ability of Q2 and Q3 was studied using NMR and UV–Vis spectroscopy. Cytotoxicity studies were carried out using the MTT assay. The influence of chelation on ROS production was studied using NMR spectroscopy in linoleic acid micelles. **Results**: It was found that only Q3 forms complexes with Fe(III) and Cu(II) ions, whereas Q2 does not demonstrate chelating properties. A cytotoxicity study revealed that Fe[Q3]_3_ significantly decreased the viability of lung cancer A549 cells, while Q3 and Cu[Q3]_2_ did not demonstrate cytotoxic properties in this cell line. Furthermore, the presence of Q3 lowered the rate of iron-induced lipid peroxidation in linoleic acid micelles. By contrast, Q2 did not influence the rate of lipid peroxidation, probably due to the absence of effective metal chelating ability. **Conclusions:** The high cytotoxic effects observed with the iron complex of Q3 against cancer cells in combination with a reduced rate of iron induced lipid peroxidation in the presence of Q3, make Q3 and its iron complex promising for further evaluation and use as chemotherapeutic agents in cancer.

## 1. Introduction

It is estimated that in the last few years there have been about 20 million new cases and 10 million deaths related to cancer each year [1,2,3]. The prevention and treatment of cancer is a major challenge for many investigators worldwide. Hundreds of experimental anticancer drugs are designed and tested because of the low rate of success with current therapies for many types of cancer [4].

Anthraquinone derivatives, such as doxorubicin, daunorubicin, and mitoxantrone, are widely used as chemotherapeutic agents in anticancer therapy [5]. They are known as DNA intercalating agents and inhibitors of DNA topoisomerase II [6,7,8,9]. An additional mechanism of the anti-proliferative activity of anthraquinones has been proposed, which is based on their ability to react with biological electron donor molecules forming a semiquinone radical. The semiquinone radical could subsequently react with oxygen, generating reactive oxygen species (ROS), as shown in Equations (1)–(4) [10,11,12,13,14,15,16].

Q + e → Q^•−^ (1)

Q^•−^ + O_2_ ↔ Q + O_2_^•−^ (2)

(3) $${O_{2}}^{•-}+{O_{2}}^{•-}\;\overset{2H^{+}}{\to }H_{2}O_{2}+O_{2}$$

O_2_^•−^ + H_2_O_2_ → O_2_ + HO^−^ + HO^•^ (4)

where, in Equations (1)–(4), Q is an anthraquinone derivative, Q^•−^ is semiquinone radical anion, e is electron, O_2_^•−^ is superoxide anion, HO^•^ is hydroxyl radical, and H_2_O_2_ is hydrogen peroxide.

The ability to generate ROS, which could be involved in the mechanism of anthraquinone’s anticancer activity has been confirmed in many studies. It was found for example, that anthraquinones could induce ROS formation in living cells [17,18,19,20,21,22,23]. In the case of the anthraquinone, purpurin, in particular, it was demonstrated that it induces ROS-associated oxidative stress in A549 cells leading to apoptosis [18]. Similarly, other anthraquinone derivatives are also able to inhibit colon cancer cell proliferation through ROS-mediated activation of the JNK signaling pathway [24]. Furthermore, it has also been shown, that the ROS formed in reactions of anthraquinones with NADPH-cytochrome P-450 could induce DNA scission [23,25].

However, the generation of ROS by anthraquinones is also considered to be one of the main mechanisms of their cardiotoxic side effects [26,27,28,29,30,31]. In particular, doxorubicin could affect the rate of lipid oxidation in cardiomyocytes, which results in ferroptosis, a programmed cell death process involving iron [28,29]. It has also been reported that carminomycin, epirubicin, idarubicin, and mitoxantrone demonstrate differential cytotoxic activity against rat cardiomyocytes, with the level of cytotoxicity in each case to be correlated to the lipophilicity of the drug [32]. By contrast, under different experimental conditions using cardiac mitochondria, it has been demonstrated that some anthraquinone derivatives could inhibit lipid peroxidation [33]. Thus, the influence of the anthraquinone derivatives on the rate of lipid peroxidation is also crucial for understanding their possible toxic side effects.

Another parameter affecting the rate of ROS production in biological systems is the presence of trace amounts of transition metal ions, especially iron and copper. These two metals can react directly with hydrogen peroxide (H_2_O_2_) via the Fenton reaction to produce highly reactive hydroxyl radicals (^•^OH). Furthermore, iron and copper chelating drugs, as well as other naturally occurring or synthetic chelators have been shown to modulate the catalytic activity of these two transition metals and can increase or decrease the rate of hydroxyl radical and ROS production [34,35,36,37,38,39,40]. In particular, some of these chelators have been shown to be both effective chelators and potent antioxidants inhibiting the Fenton reaction [41]. In this context, some anthraquinone derivatives have also demonstrated chelating ability for transition metals affecting redox activity [19,27,42,43,44]. Moreover, it appears that the reaction of the semiquinone radical anions with transition metal ions such as iron could enhance ROS generation by cyclization of the Fenton reaction as shown in Equations (5) and (6) [10,45].

Q^•−^ + Fe(III) → Q + Fe(II) (5)

H_2_O_2_ + Fe(II) → HO^−^ + HO^•^ + Fe(III) (6)

where, in Equations (5) and (6), Q is an anthraquinone derivative, Q^•−^ is a semiquinone radical anion, HO^•^ is a hydroxyl radical, and H_2_O_2_ is hydrogen peroxide.

Therefore, it could be suggested that the chelating ability of anthraquinone derivative could in general enhance ROS generation in the presence of transition metal ions. However, some chelators have also demonstrated antioxidant properties in lipid peroxidation reactions due to the reduced availability or redox activity of transition metal ions in chelate form complexes [29,46,47,48,49].

In the present study we have investigated the antioxidant/pro-oxidant properties of two novel anthraquinone derivatives in a model lipid peroxidation reaction. The lipid peroxidation in the presence of hydrogen peroxide and ferrous (Fe^2+^) ions can be described by Equations (7)–(9) [50,51,52,53,54]. The source of the ^•^OH radicals shown in Equation (7), is from the Fenton reaction described in Equation (6).

LH + ^•^OH → L^•^ + H_2_O L^•^ + O_2_ → LOO^•^ (7)

LOO^•^ + LH → LOOH + L^•^ LOOH → LO^•^ → epoxides, hydroperoxides, aldehydes (8)

L^•^ + L^•^ → L-L LOO^•^ + L^•^ → LOOL LOO^•^ + LOO^•^ → LOOL + O_2_ (9)

Here LH is a lipid molecule, L^•^ is a lipid radical, LOO^•^ is a lipid peroxyl radical, LO^•^ is a lipid alkoxyl radical, LOOH is a lipid hydroperoxide, and LOOL and L-L are covalently linked lipid dimers.

Lipid peroxidation is a chain reaction, which starts with initiation (7) and formation of lipid radicals and lipid peroxyl radical. Then, it goes to a propagation stage (8) with cyclic formation of lipid radicals and, finally, to a termination stage (9) with the formation of non-radical products.

Lipid peroxidation in the presence of chelating agents is strongly influenced by the redox-potential of metal complexes, in which case the lower the redox potential, the higher rate of peroxidation [29,55,56].

**Figure 1:**
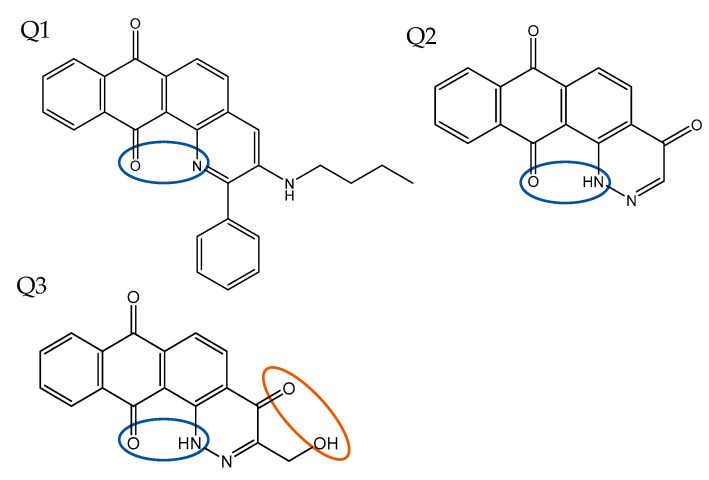
The structures of the anthraquinones 2-phenyl-4-(butylamino)naphtho[2,3-*h*]quinoline-7,12-dione (Q1), 4-hydroxynaphto[2,3-*h*]cinnoline-7,12-dione (Q2) and 3-(hydroxymethyl)naphto[2,3-*h*]cinnoline-4,7,12(1*H*)-trione (Q3). The chelating sites are marked with an oval shape drawing.

Taking into account the biological activity of the clinically used anthraquinone derivatives described above, it should be noted that the search for new compounds possessing cytotoxic properties against cancer cells and reduced toxic side effects in normal cells continues to be a major target for anticancer therapy. In this context, any new data on the structure–activity correlation for anthraquinone derivatives could help in the development of new, improved drugs. In the present work, we have studied the chelating ability of the two novel anthraquinone derivatives 4-hydroxynaphto[2,3-*h*]cinnoline-7,12-dione (Q2) and 3-(hydroxymethyl)naphto[2,3-*h*]cinnoline-4,7,12(1*H*)-trione (Q3) (Figure 1), as well as the influence of these anthraquinones and their chelate complexes with copper and iron ions on the rate of lipid peroxidation reaction. In previous studies using a similar compound, 2-phenyl-4-(butylamino)naphtho[2,3-*h*]quinoline-7,12-dione (Q1, Figure 1), it was found that the rate of lipid peroxidation decreases in the presence of its complex with iron ions, whereas it increases in the presence of its complex with copper ions [42]. In this work, a cytotoxicity study was also performed using the lung cancer cell line A549, in order to evaluate the effects of Q2 and Q3, and their iron and copper complexes on cell viability.

## 2. Results and Discussion

### 2.1. Tautomerism and Other Structural Features of the Quinone Derivatives

**Figure 2:**
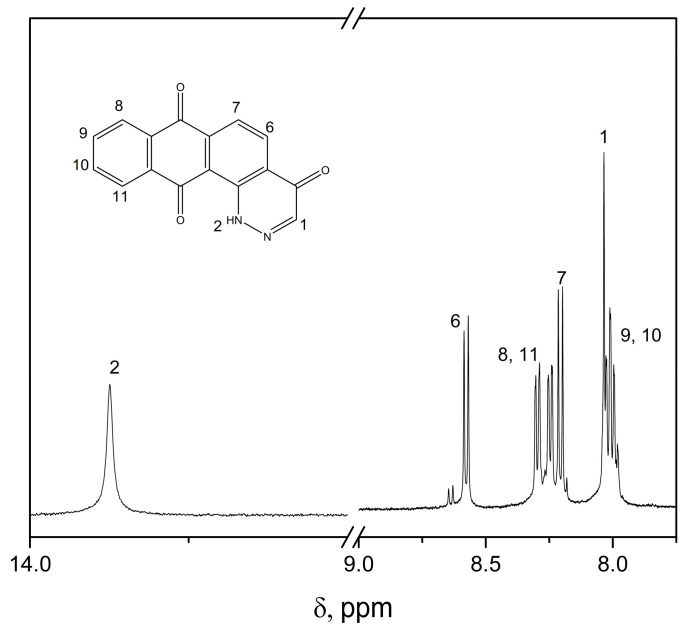
^1^H NMR spectra of 4-hydroxynaphto[2,3-*h*]cinnoline-7,12-dione (Q2) in DMSO-d6. The signals between the chemical shift (δ) 8 and 8.6 ppm belong to aromatic protons of the quinone, and the broad signal at 13.9 ppm corresponds to an -NH proton (2-H). Deuterated solvent was used as a standard. Details on spectral parameters are given in Section 3.2.

A number of studies and different methods have been used to evaluate and compare the structural and other molecular characteristics of the two quinone derivatives, Q2 and Q3. In particular, it appears from the structural features of the quinone derivatives that different tautomeric forms might exist in solution. In this context, ^1^H and ^13^C nuclear magnetic resonance (NMR), ^1^H-^1^H NMR correlation spectroscopy (COSY) and ^13^C-^1^H heteronuclear single quantum coherence (HSQC) techniques were used to elucidate the presence of the predominant tautomeric forms of the quinones Q2 and Q3 in a DMSO solution. Figure 2 represents the ^1^H NMR spectrum of the quinone Q2 in DMSO. The presence of the chemical shift signal at 13.9 ppm indicates that the predominant form of Q2 contains an NH group at the chelate center linked by a hydrogen bond with oxygen of the nearest C=O group.

**Figure 3:**
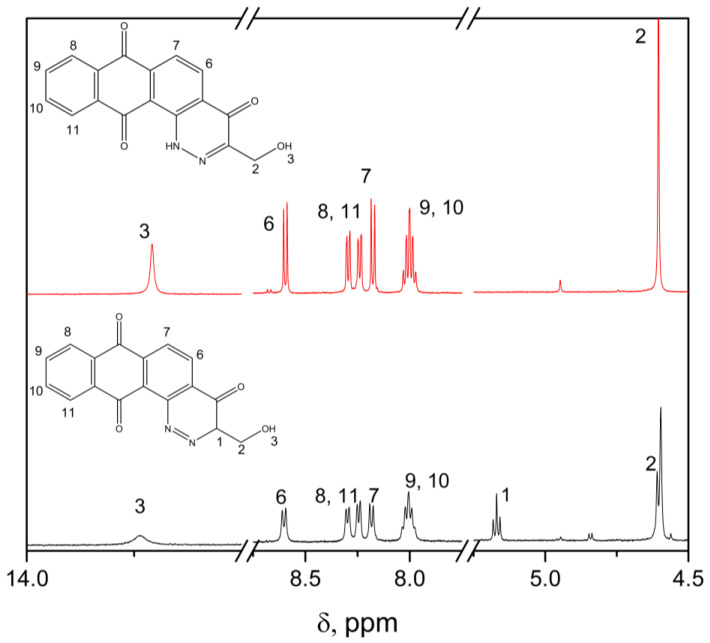
^1^H NMR spectra of 5 mM 3-(hydroxymethyl)naphto[2,3-*h*]cinnoline-4,7,12(1*H*)-trione (Q3) (top) and 1 mM Q3 (bottom) in DMSO-d6. The signals between the chemical shift (δ) 8 and 8.6 ppm belong to the aromatic protons of the quinone, the signals between 4.6 and 5.2 ppm belong to aliphatic protons of the quinone and the broad signal at 13.4 ppm corresponds to -OH proton. The assignment of NMR signals was performed according to the data obtained by the two-dimensional COSY and HSQC experiments. Deuterated solvent was used as a standard. Details on spectral parameters are given in Section 3.2.

Similar investigations were carried out for the quinone derivative Q3. Figure 3 represents the ^1^H NMR spectrum of the quinone Q3 in DMSO. As can be seen from Figure 3, at low concentrations (1 mM), the chemical shift signal at 4.6 ppm appears as an overlap of two signals; while, at high concentrations, it appears as one singlet line. Also, at low concentrations, a triplet signal at 5.17 ppm appears, which is not observed at high concentrations. This observation might be due to the presence of different tautomeric forms with different solubility. At high concentrations, the equilibrium appears to be shifted to only one of the tautomers, also due to probable differences in solubility. To test this hypothesis, the COSY and HSQC techniques were applied to elucidate the structure of the observed tautomers.

**Figure 4:**
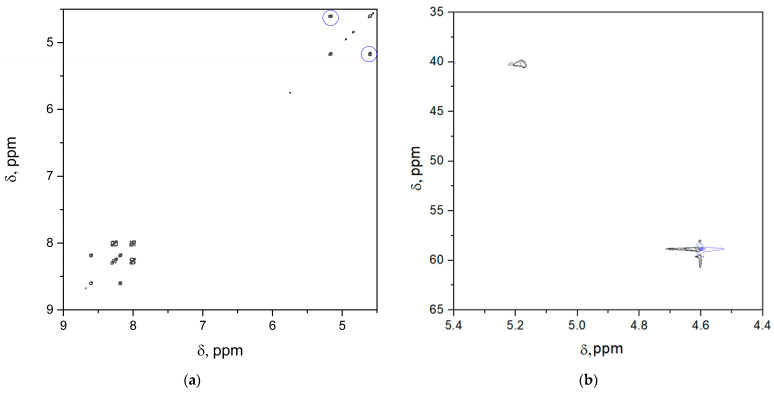
(**a**) ^1^H-^1^H NMR correlation spectroscopy (COSY) spectrum of 3-(hydroxymethyl)naphto[2,3-*h*]cinnoline-4,7,12(1*H*)-trione (Q3) in DMSO-d6. The circles indicate cross-peaks between 1-H and 2-H protons of Q3. (**b**) ^13^C-^1^H heteronuclear single quantum coherence (HSQC) spectrum of Q3 in DMSO-d6. The chemical shift (δ) is shown in the horizontal and vertical axes.

The results of these further studies appear in Figure 4a,b. Figure 4a represents the COSY spectrum and Figure 4b represents the HSQC spectrum of Q3 in DMSO. The COSY cross-peaks are observed for signals at 4.6 and 5.17 ppm at low Q3 concentration. Also, the HSQC spectrum indicates that the proton corresponding to the signal at 5.17 ppm is attached to a carbon atom. This indicates that, at low Q3 concentrations, two tautomeric forms co-exist, as suggested in Figure 3. The disappearance of the triplet signal at 5.17 ppm and the absence of the splitting for the signal of the CH_2_ group at high concentration suggest that one tautomeric form of Q3 is predominant under these conditions (Figure 3, top structure). It is notable that in methanol this tautomeric form of Q3 is the only one observed also at low concentrations, probably due to significantly lower solubility of this tautomer of Q3 in methanol.

### 2.2. The Iron and Copper Chelating Properties of the Quinone Derivatives

**Figure 5:**
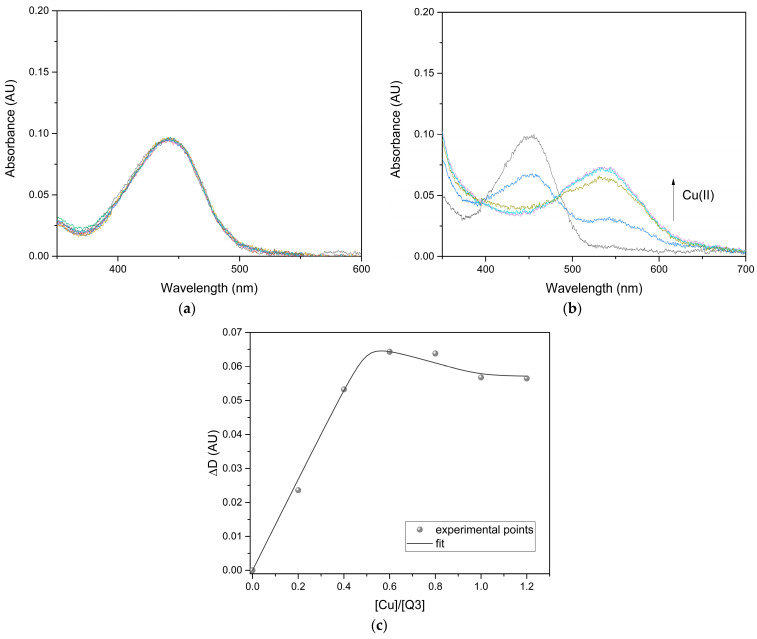
The UV–Vis absorption spectra changes of the quinones Q2 and Q3 with cupric copper in ethanol. The UV–Vis absorption spectra of (**a**) 5 × 10^−5^ M 4-hydroxynaphto[2,3-*h*]cinnoline-7,12-dione (Q2) mixed with CuCl_2_ in in the concentration range 0.5–1 × 10^−4^ M in ethanol and (**b**) 5 × 10^−5^ M 3-(hydroxymethyl)naphto[2,3-*h*]cinnoline-4,7,12(1*H*)-trione (Q3) mixed with CuCl_2_ in the concentration range 0.5–1 × 10^−4^ M in ethanol. (**c**) the dependence of the difference of absorbance of the pure chelator and its complex at 530 nm (∆D) on the concentration ratio Cu(II)/Q3. Each point represents a different experiment, and the solid line is the fitting of the points. AU is absorbance units. Further information on the fitting details is provided in Section 3.

The chelating ability of Q2 and Q3 to form complexes with iron and copper ions was studied using ultraviolet–visible (UV–Vis) and NMR spectroscopy. Figure 5 shows the UV–Vis absorption spectra of Q2 and Q3 (5 × 10^−5^ M) in ethanol in the presence of Cu^2+^ ions (concentration range 1–10 × 10^−5^ M). In the case of Q2 no changes in the absorption spectra were observed (Figure 5a). The same results were observed in the presence of the Fe^3+^ ions. By contrast, in the case of Q3, with increasing Cu^2+^ concentration, a decrease in the absorption at 450 nm and an increase in the absorption at 530 nm were observed. The dependence of the difference of absorbance of the pure chelator and its metal complex at 530 nm (∆D) is presented in Figure 5c. Similar changes were previously observed for complexes of the quinone Q1 with these metal ions [19,42]. By contrast, no changes in the absorption spectrum were observed when ferrous (Fe^2+^, FeSO_4_) or ferric (Fe^3+^, FeBr_3_) ions were added to the Q3 solution. Furthermore, no changes in the absorption spectra in DMSO solution were observed for both Q2 and Q3 in the presence of these metal ions.

The presence of isosbestic points and the saturation of ∆D at the metal–chelator ratio 0.5 indicates that the Cu(II) complex in the case of Q3 has the stoichiometry 2:1 (chelator–metal). The complex stability constants and extinction coefficients of the copper complexes of Q3 obtained from the fitting of experimental points are presented in Table 1.

K_1_ and K_2_ are stability constants of complexes of 1:1 and 2:1 (chelator–metal) molar ratio of Q3 and Cu(II) ions and where ε_1_ and ε_2_ are the corresponding extinction coefficients. Further information is provided in Section 3.

**Figure 6:**
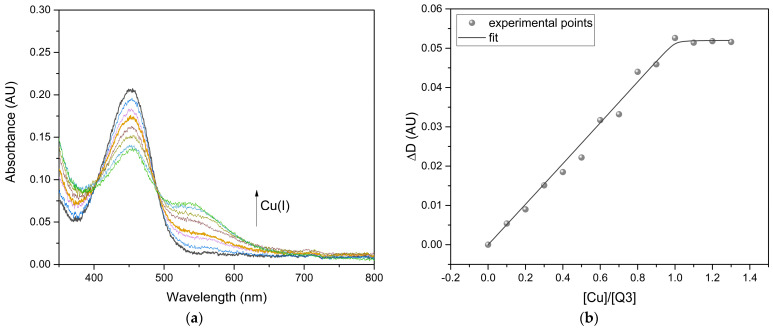
The UV–Vis absorption spectra changes of the quinone Q3 with cuprous ions in ethanol. The UV–Vis absorption spectra of 1 × 10^−4^ M 3-(hydroxymethyl)naphto[2,3-*h*]cinnoline-4,7,12(1*H*)-trione (Q3) with cuprous chloride (CuCl) in the concentration range 0.1–1.3 × 10^−4^ M in ethanol is shown in (**a**). The dependence of the difference of absorbance of pure chelator and complex at 530 nm (∆D) on the concentration ratio Cu(I)/Q3 is shown in (**b**), where each point represents a different experiment and the solid line a fit of the points. AU is absorbance units. Further information on the fitting details is provided in Section 3.

The complex formation of Q3 with Cu(I) ions (CuCl) was also studied in ethanol using UV–Vis spectroscopy. In this case, a decrease in absorbance at 450 nm and an increase of absorbance at 550 nm with increasing Cu(I) concentration is observed (Figure 6). The presence of isosbestic points and the saturation of ∆D at the chelator–metal ratio is approximately equal to 1, indicating that the cuprous ion complex has the stoichiometry 1:1 (chelator–metal).

The stability constants and extinction coefficients of the complex of Q3 with cuprous ion Cu(I) obtained from the fitting of experimental points are presented in Table 2.

K_1_ is the stability constant of the Q3 complex with cuprous ions Cu(I), at 1:1 (chelator–metal) ratio and ε1 is the corresponding extinction coefficient in ethanol. Further information is provided in Section 3.

**Figure 7:**
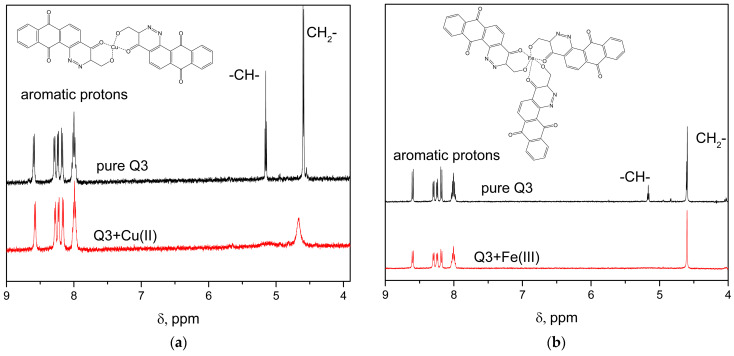
(**a**) ^1^H NMR spectra of 2 mM 3-(hydroxymethyl)naphto[2,3-*h*]cinnoline-4,7,12(1*H*)-trione (Q3) in DMSO in the absence of metal ions and in the presence of 1 mM cupric chloride (CuCl_2_), (**b**) ^1^H NMR spectra of 0.5 mM 3-(hydroxymethyl)naphto[2,3-*h*]cinnoline-4,7,12(1*H*)-trione (Q3) in DMSO in the absence of metal ions and in the presence of 0.17 mM Fe(NO_3_)_3_ and the possible structure of the [Cu(Q3)_2_]-complex in DMSO (The chemical shift (δ) is shown in the horizontal axis). Deuterated solvent was used as a standard.

The results obtained in this study indicate that the interaction of Q3 with Cu^2+^ and Fe^3+^ ions in DMSO, as well as with Fe^3+^ in ethanol, does not affect the transitions in the π-system of the quinone. Q3 potentially contains two chelating centers, as shown in Figure 1, one in the aromatic part of the molecule and one in the aliphatic part; while Q2 contains only one chelate center, which is located in the aromatic part. UV–Vis spectroscopy data for Q2 indicate that this quinone derivative does not demonstrate metal chelating ability. Furthermore, additional NMR experiments were carried out, due to the possibility that Q3 may form complexes via another chelate center. In this context, the ^1^H NMR spectra of Q3 in the presence and in the absence of cupric ions (CuCl_2_) in DMSO is shown in Figure 7a. It is notable that, in the presence of Cu(II) ions, significant broadening is observed for the signals of CH_2_ and CH protons of the Q3 side chain. This broadening appears to be caused by the close position of these protons to the metal ion in the chelate center. At the same time, no broadening was observed for the aromatic protons of Q3. This finding suggests that the metal complex formation in DMSO happens via interactions with oxygen (Figure 7a). The same results were obtained in the interaction of Q3 with ferric ions Fe^3+^ (Fe(NO_3_)_3_, Figure 7b). The stoichiometry of complexes was determined by the determination of the “saturation” metal concentration, after which no changes in the spectra were observed. For Cu(II)-Q3, the stoichiometry (metal–chelator) was 1:2, and for Fe(III)-Q3 the stoichiometry (metal–chelator) was 1:3. Schematic structure of the complexes with these stoichiometries are shown in Figure 7.

**Figure 8:**
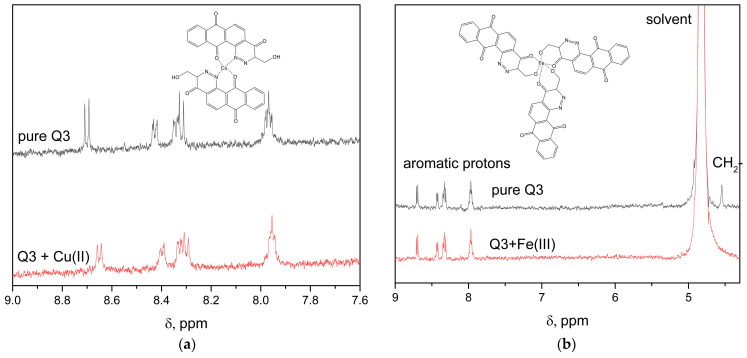
^1^H NMR spectra of 0.5 mM 3-(hydroxymethyl)naphto [2,3-*h*]cinnoline-4,7,12(1*H*)-trione (Q3) in CD_3_OD (**a**) in the absence and in the presence of 0.25 mM cupric chloride (CuCl_2_), (**b**) in the absence and in the presence of 0.17 mM Fe(NO_3_)_3_ (The chemical shift (δ) is shown in the horizontal axis). Deuterated solvent was used as standard.

**Figure 9:**
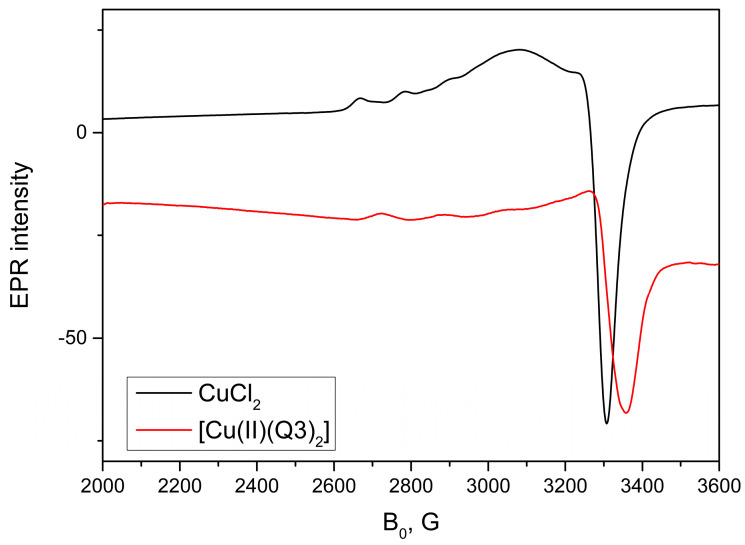
EPR spectra of CuCl_2_ and Cu(Q3)_2_ in DMSO at 100 K.

These findings suggest that the metal complex formation in DMSO takes place via the chelate center containing the OH-group. Furthermore, it explains the absence of changes in the UV–Vis spectra since, in this case, the π-system transitions are not affected. At the same time, changes to the UV–Vis absorption spectra in ethanol means that the complex formation takes place via a chelate center containing an NH group (Figure 8), affecting the π-system transitions. The NMR spectra of the mixture of Q3 (0.5 mM) and CuCl_2_ (0.25 mM) in deuterated methanol are shown in Figure 8. In contrast to DMSO, line broadening and shift of aromatic protons of Q3 is observed in the presence of CuCl_2_, indicating that cupric copper ions are placed closer to the aromatic fragment. Additional experiments were performed to confirm the oxidation state of copper using electron paramagnetic resonance (EPR). The EPR spectra of CuCl_2_ and [Cu(Q3)_2_] complex in DMSO are given in Figure 9. The characteristic signal of Cu(II) was observed in the complex. The difference between the EPR spectrum of the copper (II) salt and the copper(II) complex with the chelator Q3 can be shown, since the linewidth and g-value are clearly different. The change of these magnetic parameters is caused by the influence of the chelators on the electronic structure of the copper(II) ion [57,58]. The presentation of iron ions in the EPR spectrum is often problematic because of the short electron spin relaxation time by these ions. In this context, fast spin relaxation translates into broadening of the EPR line and a corresponding decrease of its intensity, which is inversely proportional to the square of the linewidth.

The NMR spectra of the mixture of Q3 (0.5 mM) and Fe(NO_3_)_3_ (0.17 mM) in deuterated methanol are shown in Figure 8. As with the DMSO, line broadening of CH_2_ protons of aliphatic fragment of Q3 is observed in the presence of Fe(NO_3_)_3_, indicating that cupric copper ions are placed closer to the aliphatic fragment. The signal corresponding to CH protons (H-2) are not observed due to overlapping with the water signal from CD_3_OD.

The ability of Q2 to form chelate complexes with metal ions was also tested by means of NMR spectroscopy. Figure S1 represents the ^1^H NMR spectra of 0.5 mM Q2 at concentrations of CuCl_2_ in the range 0.05–1 mM in CD_3_OD. It appears that no significant changes of the NMR spectrum of Q2 were observed with increasing the Cu(II) concentration. These results, together with the absence of the changes to the UV–Vis spectra of Q2 in the presence of copper ions, suggest that Q2 does not have the ability to form complexes with copper ions. The same result was obtained with iron ions.

### 2.3. Cytotoxic Activity of the Quinones Q2 and Q3 and Their Iron and Copper Complexes

Preliminary investigations to examine the anticancer and other possible toxic side effects of the quinones Q2 and Q3, as well as their iron and copper metal complexes in cells were also carried out. In particular, the cytotoxic effects of these quinones and their metal complexes were studied in cell cultures using A549 cells, a human lung carcinoma cell line. The cytotoxic effects of the quinones and their metal complexes were compared to untreated A549 cells (controls) under the same conditions. In each case, cell viability was measured using the 3-(4,5-dimethylthiazol-2-yl)-2,5-diphenyltetrazolium bromide (MTT) assay method [59].

**Figure 10:**
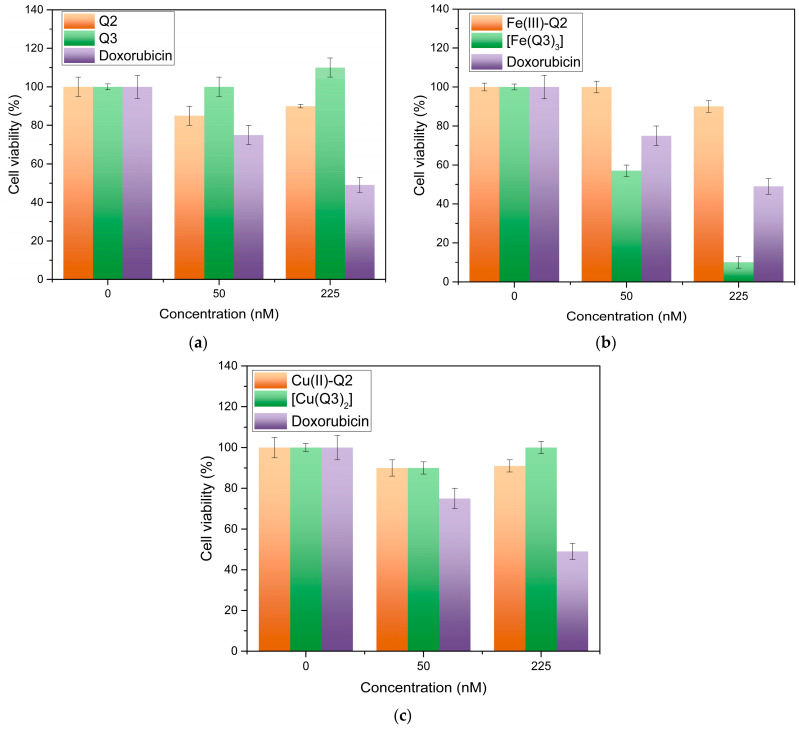
Cytotoxic studies of the quinones and their metal complexes using a cancer cell line. Viability of A549 cells in the presence of the quinones Q2 and Q3 and their iron and copper complexes. The cells were incubated for 48 h with (**a**) 4-hydroxynaphto[2,3-*h*]cinnoline-7,12-dione (Q2), 3-(hydroxymethyl)naphto[2,3-*h*]cinnoline-4,7,12(1*H*)-trione (Q3), and the complexes with (**b**) ferric ions (Fe(III)) and (**c**) cupric ions (Cu(II)). Each value represents the mean ± SD.

The results of the cell studies using the quinones Q2 and Q3, as well as their iron or copper complexes are represented in Figure 10. Doxorubicin was used as a positive control. It appears that both the quinones, as well as their complexes with copper ions have not caused a significant decrease in cell viability (Figure 10 a,c). However, under the same conditions, it appears that only the ferric complex of Q3 ([Fe(Q3)_3_]) induces a significant decrease in cell viability; whereas, by contrast, the mixture Fe(III)-Q2 has no effects under the same conditions (Figure 10b).

### 2.4. Iron- and Copper-Mediated Lipid Peroxidation

**Figure 11:**
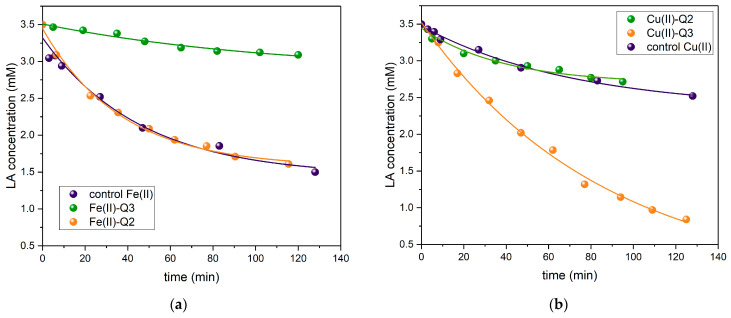
Lipid peroxidation studies in the presence of the quinone chelators and their metal complexes. Kinetics of the (**a**) ferrous ion iron (Fe(II)) and (**b**) cupric (Cu(II)) copper-mediated initiation reaction of LA peroxidation in the absence and presence of the 4-hydroxynaphto[2,3-*h*]cinnoline-7,12-dione (Q2) and 3-(hydroxymethyl)naphto[2,3-*h*]cinnoline-4,7,12(1*H*)-trione (Q3) at 25 °C (see Section 3 for details). Each point represents one experiment, and the solid line corresponds to the exponential fitting.

The redox properties of the quinones Q2 and Q3 were studied by monitoring the rate of lipid peroxidation in a model system of linoleic acid micelles (LA micelles), using NMR spectroscopy as previously described [42,60,61]. In brief, the dependence of intensity of the NMR signal corresponding to the bis-allylic proton of LA in the reaction mixture with time was recorded. The decrease of the intensity of this signal corresponds to hydrogen atom detachment from the initial LA at the initiation stage of LA peroxidation (Equation (7)). In these studies, the reaction mixture contained 3.5 mM of LA in PBS, 0.1 mM of FeSO_4_, 0.5 M of H_2_O_2_, and 0.5 mM of quinone. The resulting kinetics are shown in Figure 11a. The samples without the presence of the quinone molecules were considered as the controls. As it can be seen in Figure 11a, no changes in the LA peroxidation rate were observed in the presence of Q2 in comparison with the control sample. At the same time, Q3 significantly reduced the rate of LA peroxidation under the same conditions. It should be noted that similar results were observed in earlier studies with the quinone Q1 [42]. The absence of influence on lipid peroxidation by Q2 is probably related to the absence of chelating ability by this quinone.

The kinetics of cupric copper-mediated LA peroxidation in the presence of Q2 and Q3 are shown in Figure 11b. It also appears in this case that there is no effect of Q2 on the rate of lipid peroxidation, which is similar to the case of ferrous iron ions. By contrast, Q3 significantly increased the rate of LA oxidation in the presence of cupric copper ions. The same result was previously observed using Q1 [42]. The estimation of the rate constants of the reactions are given in Table 3.

Considering the difference in redox activity of the iron and copper complexes of Q3, it can be assumed that, after the addition of the mixture of ferrous iron (Fe^2+^) and H_2_O_2_ (Fenton reagents) to the suspension of LA micelles, fast formation of less active oxidized ferric (Fe^3+^) ions are formed, which are less active than Fe^2+^ ions for the Fenton reaction in the presence of hydrogen peroxide. The Fe^3+^ ions react with Q3 causing the formation of a redox inactive complex with three Q3 ligands, as shown in the previous sections.

By contrast, the reaction of cupric ions (Cu^2+^) with H_2_O_2_ results in the formation of a Cu^+^ ion. In this case, it can be assumed that Q3 forms a redox active complex with Cu^+^ ions, in which case the metal ion is easily accessible to the H_2_O_2_ molecule for proceeding with the Fenton-like reaction.

Overall, these data related to the molecular differences of the quinones and their iron and copper complexes might be useful for the design of new, more specific anti-cancer drugs. For example, similar molecular mechanisms for the cardiotoxicity of anthraquinone derivatives has been proposed, which is attributed to their ability to form ROS, which in turn are thought to induce damage to cardiomyocyte membranes as a result of lipid peroxidation [26,27,28,29]. Furthermore, it has also been previously shown that the formation of complexes of anthraquinone derivatives with metal ions could also lead to either an increase or a decrease of redox properties [29,46].

According to the data of the ^1^H NMR and UV–Vis spectroscopy described in Section 2.2, the novel anthraquinone derivative Q3 has demonstrated the presence of metal chelating potential. Furthermore, the use of these techniques allowed the identification of changes in the metal complex structure in different solvents. By contrast, in the case of the other novel anthraquinone derivative Q2, no chelating ability was found, despite the structural similarity of Q2 and Q3. The ^1^H NMR technique has also allowed the characterization of the influence of the quinone derivatives Q2 and Q3 on the rate of metal-induced lipid peroxidation. In this case, it was found that, unlike Q2, which does not affect the rate of lipid peroxidation, Q3 has shown a significant influence on the rate under similar conditions. These changes may be attributed to the differences in chelating ability of these compounds. Overall, it is suggested that the chelating ability of the anthraquinone derivatives may play an important role in terms of their cardiotoxicity.

Furthermore, it was also found that Q3 significantly increases the rate of copper-induced lipid peroxidation and decreases the rate of iron-induced lipid peroxidation. Cell studies have also demonstrated that iron complexes of Q3 possess the anti-proliferative activity, while copper complexes of Q3 and the parent compound Q3 do not demonstrate cytotoxic properties in the human lung carcinoma cell line A549. Therefore, the iron complex of Q3 could be considered as a possible chemotherapeutic agent with cytotoxic properties against cancer cells and also with reduced lipid peroxidation toxicity in normal cells.

## 3. Materials and Methods

### 3.1. Materials

Doxorubicin was purchased from Sigma Aldrich (St. Louis, MO, USA). Linoleic acid (LA) > 99.0% purity was purchased from Shanghai Aladdin Bio-Chem Technology Co., Ltd., Shanghai, China. Deuterated water D_2_O (99.9% D), FeSO_4_, FeBr_3_, CuCl_2_, CuCl, and H_2_O_2_ (35.5%) were obtained from Sigma Aldrich (St. Louis, MO, USA). DMSO-d6 (99.8% D) was purchased from Carl Roth (Karlsruhe, Germany). Deuterated methanol (99.8% D) was obtained from Cambridge Isotope Laboratories (Tewksbury, MA, USA).

Chloroform was purchased from Sigma Aldrich (St. Louis, MO, USA). Anhydrous sodium sulfate was added to the chloroform solution and left for 12 h. The solution was then filtered and evaporated under reduced pressure.

Human lung carcinoma cells A549 (CCL-185) were purchased from American Type Culture Collection (ATCC, Manassas, VA, USA).

### 3.2. Q2 and Q3 Synthesis

#### 3.2.1. 1-Amino-2-(3-hydroxyprop-1-yn-1-yl)anthracene-9,10-dione

1-amino-2-iodo-9,10-anthraquinone was synthesized by the method described in reference [62] (mp 164–165 °C). A mixture of the 1-amino-2-iodo-9,10-antraquinone (0.2 g, 0.6 mmol), Na_2_CO_3_ (0.06 g, 0.6 mmol), 5 mg Pd(PPh_3_)_2_Cl_2_, 3 mg CuI, and 2-propyn-1-ol (0.04 g, 0.041 mL, 0.7 mmol) in 3.9 mL pyridine and 1.9 mL H_2_O was stirred under argon stream at 75 °C (6 h) until iodide was consumed (thin layer chromatography (TLC) control). The reaction mixture was cooled, poured into 100 mL chloroform, washed with 400 mL aqueous HCl (0.5%) and H_2_O. More chloroform (100 mL) was added. The chloroform solution was dried over sodium sulfate and filtered. Then the solvents were evaporated. The crude product was chromatographed on SiO_2_. The yield of 1-amino-2-(3-hydroxyprop-1-yn-1-yl)anthracene-9,10-dione was 0.13 g (81%). The melting point (mp) was estimated at 225–227 °C (CHCl_3_/pentane) [63].

#### 3.2.2. 4-Hydroxynaphto[2,3-*h*]cinnoline-7,12-dione (Q2, Scheme 1)

H_2_SO_4_ (10 mL) and NaNO_2_ (0.8 g, 1.2 mmol) in H_2_O (1 mL) were added successively to 1-amino-2-ethynyl-9,10-anthraquinone (0.10 g, 0.4 mmol) in acetone (25 mL). The mixture was stirred for 1 min, the solution of the formed diazo salt was poured out into 38.0% H_2_SO_4_ (100 mL) and stirred for 30 min at 65 °C. Cyclization products were extracted with chloroform. The extract was thoroughly washed with water and dried. Product crystallization of the dry residue occurred from a toluene/hexane mixture. The yield of Q2 was 0.10 g (89%), the mp was estimated to be 290–292 °C (toluene/hexane). The spectrum ^1^H NMR (DMSO-d6) showed δ (ppm): 13.87 (1H, s, 2-H), 8.58 (1H, *J* = 8.4 Hz, d, 6-H), 8.30 (1H, *J* = 7.3 Hz, d, 8-H), 8.25 (1H, *J* = 8.6 Hz, d, 11-H), 8.20 (1H, *J* = 8.4 Hz, d, 7-H), and 8.06–7.96 (3H, m, 1,9,10-H). The spectrum ^13^C NMR (DMSO-d6) showed δ (ppm): 185.6, 182.6, 170.3, 143.0, 140.5, 137.9, 135.6, 135.4, 133.8, 132.6, 132.1, 127.4, 127.2, 126.4, 121.8, and 119.6. The analytical calculation for C_16_H_8_N_2_O_3_ was C, 69.56; H, 2.92; N, 10.14. Found: C, 69.76; H, 2.97; N, 10.13. HR-ESI-MS (*m*/*z*): calculation for C_16_H_8_N_2_O_3_ [M+H^+^]^+^ was 277.061. Found: 277.060. HR-ESI-MS(−) (*m*/*z*): calculation for C_16_H_8_ N_2_O_3_ [M–H^+^]^−^ was 275.046. Found: 275.043. In the infra-red (IR) spectrum, ν (cm^−1^) were 1650, 1680 (C=O) and 3315 (NH) [64] (Figure S3a).

**Scheme 1:**
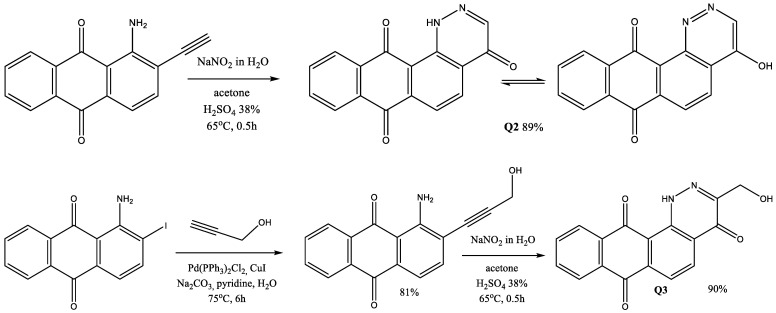
Reaction scheme of the synthesis Q2 and Q3.

#### 3.2.3. 3-(Hydroxymethyl)naphtho [2,3-*h*]cinnoline-4,7,12(1*H*)-trione (Q3, Scheme 1)

A mixture of the 1-amino-2-(3-hydroxyprop-1-yn-1-yl)anthracene-9,10-dione (0.083 g, 0.3 mmol) in acetone (19 mL) was stirred at 20 °C. An additional 9.64 g (7.3 mL 37 mmol) 38% H_2_SO_4_ and 0.062 g (0.9 mmol) NaNO_2_ in 0.75 mL water were successively added to the reaction mixture. The reaction mixture was shaken for one minute and poured into a solution of 38% H_2_SO_4_, then stirred at 65 °C (0.5 h). Cyclization products were extracted with chloroform. The products were recrystallized. The yield of 3-(Hydroxymethyl)naphtho[2,3-*h*]cinnoline-4,7,12(1*H*)-trione was 0.082 g (90%), the mp was estimated to be 233–236 °C (toluene/hexane). The ^1^H NMR (DMSO-d6) spectrum showed δ (ppm): 13.74 (1H, s, 3-H), 8.60 (1H, *J* = 8.4 Hz, d, 6-H), 8.30 (1H, *J* = 7.3 Hz, d, 8-H), 8.25 (1H, *J* = 7.0 Hz, d, 11-H), 8.19 (1H, *J* = 8.4 Hz, d, 7-H), 8.05–7.95 (2H, m, 9,10-H), 5.17 (1H, *J* = 6.1 Hz, t, 1-H), and 4.61 and 4.60 (1H, s, 2-H of various tautomeric form); The ^13^C NMR (DMSO-d6) spectrum showed δ (ppm): 185.6, 182.6, 169.6, 152.2, 140.8, 137.8, 135.6, 135.4, 133.8, 132.6, 132.4, 127.3, 127.2, 125.5, 121.4, 119.4, 58.9, and 29.4. The HR-ESI-MS(+) (*m*/*z*): calculation for C_17_H_10_N_2_O_4_ [M+H^+^]^+^ was estimated to be 307.071 and found to be 307.071 and 289.0604 (-OH group). The HR-ESI-MS(−) (*m*/*z*): calculation for C_17_H_10_N_2_O_4_ [M–H^+^]^−^ was estimated to be 305.057 and found to be 305.057. In the infra-red (IR) spectrum, ν (cm^−1^) were 1664 and 1680 for (C=O) and 3317 for (NH) (Figure S3b).

The melting points were measured on Kofler hot-plate apparatus HMK (Franz Küstner Nacht KG, Dresden, Germany). The IR spectra were recorded with a Shimadzu IRTracer-100 instrument with GS10802-B Quest ATR ZnSe Accessory (Specac, Orpington, UK). The UV–Vis absorption spectra were recorded with SF2000 spectrophotometer (Spectrum, Moscow, Russia). Elemental analysis was conducted using CHNS analyzer EA-3000 (EuroVector, Pavia, Italy).

Thin-layer chromatography (TLC) was conducted using Silica gel 60 F254 plates (Merck 1.05554.0001; Darmstadt, Germany), Q2 Rf 0.69 (EtOAc/CH_2_Cl_2_—1:1), Q3 Rf 0.62 (EtOAc).

Molecular formulae were additionally confirmed by HR-ESI-MS, using the coincidence of theoretical and experimental exact masses and relative abundances for all isotopes in isotopic distributions (see Figure S1 in the Supplementary Materials).

An outline of the reactions and conditions for the synthesis of the anthraquinones derivatives 4-hydroxynaphto[2,3-*h*]cinnoline-7,12-dione (Q2) and 3-(hydroxymethyl)naphtho[2,3-*h*]cinnoline-4,7,12(1*H*)-trione (Q3) are shown in Scheme 1.

### 3.3. NMR Study

The ^1^H NMR spectra were recorded on a Bruker AVHD-500 (500 MHz) NMR spectrometer (Bruker, Billerica, MA, USA) using a temperature of 300 K. The 2D ^1^H-^1^H COSY experiment was carried out using gradient and purge pulses (cosygpppqf pulse sequence), using 5 s relaxation delay. The HSQC spectrum was recorded with phase-sensitive ge-2D multiplicity-edited HSQC using echo–antiecho and adiabatic pulses (hsqcedetgpsp) [65,66], using 5 s relaxation delay, 3.6 ms evolution time.

### 3.4. EPR Study

EPR measurement was carried out on X-band ELEXSYS ESP-580E EPR spectrometer equipped with cylindrical EPR cavity ER 4118X-MD5 at 100 K. Microwave power was 6 mW. Modulation amplitude was 3 G.

### 3.5. High-Resolution Electrospray Mass Spectrometric Study

The high-resolution electrospray mass spectrometric (HR-ESI-MS) analysis was performed at the Center of Collective Use «Mass spectrometric investigations» SB RAS with a direct injection of liquid samples via an automatic syringe pump to an electrospray ionization quadrupole time-of-flight (ESI-Q-TOF) mass spectrometer Maxis 4G (Bruker Daltonics, Bremen, Germany). Mass spectra were recorded in both positive and negative modes within 50–1000 *m*/*z* range.

### 3.6. Chelate Metal Complex Stability Constants Determination

The calculation of the chelate complex parameters was carried out on the basis of the following model of complex formation [19,60]:

(10) $$Q+M\overset{K_{1}}{↔}QM$$

(11) $$QM+M\overset{K_{2}}{↔}Q_{2}M$$

where Q is the quinone molecule, M is the metal ion, and K_1_ and K_2_ are the stability constants of the complexes 1:1 and 2:1 (chelator–metal), correspondingly.

The method of calculation of the stability constants K_1_ and K_2_ and the extinction coefficients ε_1_ and ε_2_ for 1:1 and 2:1 molar ratio chelator–metal complexes, respectively, has been carried out as previously described [19,60]. In brief, the dependencies of ∆D (the change of the chelator absorption on a fixed wavelength) on metal concentration have been determined. The analytical expression for ∆D in this model has been previously described [19,60] as follows:

∆D = ε_0_[Q] + ε_1_ K_1_[Q][M] + ε_2_ K_1_K_2_[Q]_2_[M] − ε_0_ C_Q_, (12)

where ε_0_, ε_1_, and ε_2_ are the extinction coefficients of the chelator, the metal complex of the 1:1 and the complex of the 2:1 molar ratio, respectively; K_1_ and K_2_ are stability constants of the complex 1:1 and the complex 2:1 molar ratio, respectively; and C_Q_ is the initial quinone concentration. The stability constants and extinction coefficients were obtained from fitting the parameters using the Levenberg–Marquardt algorithm using the Python script.

### 3.7. Sample Preparation for the Lipid Peroxidation Studies

The reaction mixtures for the ^1^H NMR studies consisted of LA micelles, with LA concentration of 3.5 mM. In the reactions with hydrogen peroxide, 0.5 M H_2_O_2_ was added with 0.1 mM of FeSO_4_ or CuCl_2_ freshly prepared in PBS (pH 7.4). For the studies examining the effects of Q2 and Q3 on LA oxidation via the Fenton reaction, the quinone was added to the lipid solution in chloroform before drying and removal of the solvent and subsequent hydration in PBS (pH 7.4) [60,61]. The final concentration of the chelator was 0.5 mM.

### 3.8. Cytotoxicity Study

The 3-(4,5-dimethylthiazol-2-yl)-2,5-diphenyltetrazolium bromide (MTT) assay was used to compare the cytotoxic effects of the different mixtures and compare them to the control samples [67]. The samples tested were doxorubicin, quinone Q2, and its mixtures with iron (Fe(III)) and copper (Cu(II)), as well as quinone Q3 and its chelate metal complexes (Fe[Q3]_3_) and Cu[Q3]_2_.

The cytotoxicity study involved the following procedure. The A549 cells were seeded in a 96-well plate at a density of 3500 cells/well in 100 μL of Dulbecco’s Modified Eagle Medium (DMEM) supplemented with 10% PBS, 2 mM L-glutamine, antibiotic–antimycotic mix, and incubated for 24 h. Subsequently, 100 μL of DMEM containing quinones or quinone metal complexes at quinone concentrations of 150–250 nM were added to each well. The cells were then incubated at 37 °C for 48 h. Following incubation, the medium was removed, and the cells were incubated with 150 μL DMEM containing 0.35 mg/mL MTT for 3 h. After the incubation period, the medium was removed, and the formazan crystals were dissolved by adding 150 μL of DMSO per well. The plate was then shaken, and the absorbance values were recorded at 570 nm. Untreated cells were used as a control. Cell viability was expressed as a percentage of control ± SD.

## 4. Conclusions

The present study has examined the metal chelating and cytotoxic properties of the novel anthraquinone derivatives 4-hydroxynaphto[2,3-*h*]cinnoline-7,12-dione (Q2) and 3-(hydroxymethyl)naphto[2,3-*h*]cinnoline-4,7,12(1*H*)-trione (Q3). It has been shown that Q3 could form complexes with iron and copper ions, but the active chelate center differs in its capacity to bind these metals in different solvents. However, it appears that Q2 does not demonstrate chelating properties under the same conditions. In further studies in cells, it was revealed that the iron complex [Fe(III)[Q3]_3_] could significantly decrease the viability of the lung cancer cells, A549; while under the same conditions, the parent compound Q3 and its copper complex [Cu[Q3]_2_] could not demonstrate cytotoxic effects on this cell line. At the same time, in different studies involving hydrogen peroxide, it has been shown that the presence of Q3 lowered the rate of ferrous iron-induced lipid peroxidation in linoleic acid (LA) micelles but increased the rate in cupric copper-induced lipid peroxidation, probably due to redox cycling. Under the same conditions, Q2 did not influence the rate of lipid peroxidation, probably due to the absence of chelating ability for iron and copper.

These findings provide important information for the mode of action of the anthraquinone drugs which are widely used in cancer chemotherapy, but their use is largely limited due to their cardiotoxicity. According to recent concepts, the cardiotoxic side effects observed by the anthracycline drugs is caused by a high degree of generation of ROS, especially in the presence of iron. This, in turn, stimulates lipid peroxidation of cardiomyocyte membranes and subsequently cardiomyocyte damage. In this study, as well as in previous studies, both an increase in the rate of lipid oxidation and its inhibition may be observed for different quinone derivatives depending on their structure. In this context, the reduction of cardiac complications caused by new cancer chemotherapy drugs may allow higher doses of drugs to be used, and potentially increase the therapeutic rate in cancer. From this point of view, the effects of high cytotoxicity observed for iron complexes against cancer cells in combination with a reduced rate of lipid peroxidation, such as those observed for Q3, make this quinone and other similar drugs promising for use as chemotherapeutic agents for the treatment of cancer.

## Figures and Tables

**Table 1:** The calculated parameters of Q3 chelating complexes with cupric ions in ethanol solution.

Parameter	[Cu(II)(Q3)_2_] in Ethanol
ε_1_	1750 ± 80 M^−1^ cm^−1^
K_1_	(5 ± 1) × 10^6^ M^−1^
ε_2_	4440 ± 100 M^−1^ cm^−1^
K_2_	(7.5 ± 2.5) × 10^5^ M^−1^

**Table 2:** The calculated parameters of Q3 chelating complexes with cuprous ions in ethanol solution.

Parameter	[Cu(I)Q3]
ε1	580 ± 70 M^−1^ cm^−1^
K_1_	(3 ± 1) × 10^7^ M^−1^

**Table 3:** The rate constants for the ferrous iron- and cupric copper-mediated initiation reaction of linoleic acid peroxidation in micelles in the presence of Q2 and Q3 relative to the control.

Sample	k, s^−1^
control, Fe(II)	(3.6 ± 0.8) × 10^−4^
control, Cu(II)	(0.8 ± 0.2) × 10^−4^
Q2-Fe(II)	(4.5 ± 0.6) × 10^−4^
Q2-Cu(II)	(0.6 ± 0.3) × 10^−4^
Q3-Fe(II)	(0.2 ± 0.1) × 10^−4^
Q3-Cu(II)	(2 ± 0.4) × 10^−4^

Abbreviations: Q2: 4-hydroxynaphto[2,3-*h*]cinnoline-7,12-dione; Q3: 3-(hydroxymethyl)naphto[2,3-*h*]cinnoline-4,7,12(1*H*)-trione; k is the rate constant of the initiation stage of linoleic acid peroxidation (in s^−1^). In the controls, Fe(II) ions were added as FeSO_4_ and Cu(II) ions as CuCl_2,_ in the absence of quinones in both cases.

## Data Availability

Data are contained within the article.

## Supplementary Materials

The following supporting information can be downloaded at: https://www.mdpi.com/article/10.3390/ph17121717/s1, Figure S1: NMR studies of the interaction of Q2 with cupric ions in methanol; Figure S2: HR-ESI-MS spectra of (a) Q2 and (b) Q3; Figure S3: IR spectra of (a) Q2 and (b) Q3.

## Abbreviations

A549	Human lung carcinoma cell line
AQs	Anthraquinone derivatives
AU	Absorbance units
δ	Chemical shift
COSY	Correlation spectroscopy
DMEM	Dulbecco’s Modified Eagle Medium
DMSO	Dimethyl sulfoxide
DNA	Deoxyribonucleic acid
EPR	Electron paramagnetic resonance
HR-ESI-MS	High-resolution electrospray mass spectrometry
HSQC	Heteronuclear single quantum coherence
LA	Linoleic acid
MTT	3-(4,5-Dimethylthiazol-2-yl)-2,5-diphenyltetrazolium bromide
mp	Melting point
NMR	Nuclear magnetic resonance
PBS	Phosphate-buffered saline
ppm	Part per million
Q1	2-Phenyl-4-(butylamino)naphtho[2,3-*h*]quinoline-7,12-dione
Q2	4-Hydroxynaphto[2,3-*h*]cinnoline-7,12-dione
Q3	3-(Hydroxymethyl)naphto[2,3-*h*]cinnoline-4,7,12(1*H*)-trione
ROS	Reactive oxygen species
TLC	Thin layer chromatography
UV–Vis	Ultraviolet–visible
