# Supplementary material for: Novel Anthraquinone Derivatives and Their Complexes with Metal Ions with Anticancer Activity: Structure/Redox and Chelation Activity Correlations
Source: Pharmaceuticals (Basel). 2024 Dec 19;17(12):1717. doi: 10.3390/ph17121717 (PMC11678833; doi:10.3390/ph17121717)
Supplement: Supplementary file 1 [file pharmaceuticals-17-01717-s001.zip › pharmaceuticals-3332465-supplementary.pdf]

Supplementary information

Novel Anthraquinone Derivatives and Their Complexes with Metal Ions with Anticancer Activity: Structure/Redox and Chelation Activity Correlations

Olga Yu. Selyutina [1], Maya A. Ulyanova [1], Olga A. Chinak [1,2], Viktor A. Timoshnikov [1], Lidiya G. Fedenok [1], Alexander A. Stepanov [1], Vadim V. Yanshole [3], Leonid V. Kulik [1], Sergey F. Vasilevsky [1], Nikolay E. Polyakov [1] and George J. Kontoghiorghes [4,*]

[1] Institute of Chemical Kinetics and Combustion, Institutskaya St., 3, Novosibirsk 630090, Russia; olga.gluschenko@gmail.com (O.Y.S.); m.ulyanova1@g.nsu.ru (M.A.U.); chinakolga@gmail.com (O.A.C.); timoshnikov@kinetics.nsc.ru (V.A.T.); fedenok@kinetics.nsc.ru (L.G.F.); stepanov@kinetics.nsc.ru (A.A.S.); chemphy@kinetics.nsc.ru (L.V.K.); vasilev@kinetics.nsc.ru (S.F.V.);
polyakov@kinetics.nsc.ru (N.E.P.)

[2] Institute of Chemical Biology and Fundamental Medicine SB RAS, Lavrentyev Ave. 8, Novosibirsk 630090, Russia

[3] International Tomography Center SB RAS, Institutskaya Str. 3a, Novosibirsk 630090, Russia; vadim.yanshole@tomo.nsc.ru

[4] Postgraduate Research Institute of Science, Technology, Environment and Medicine, Limassol CY-3021, Cyprus

* Correspondence: kontoghiorghes.g.j@pri.ac.cy

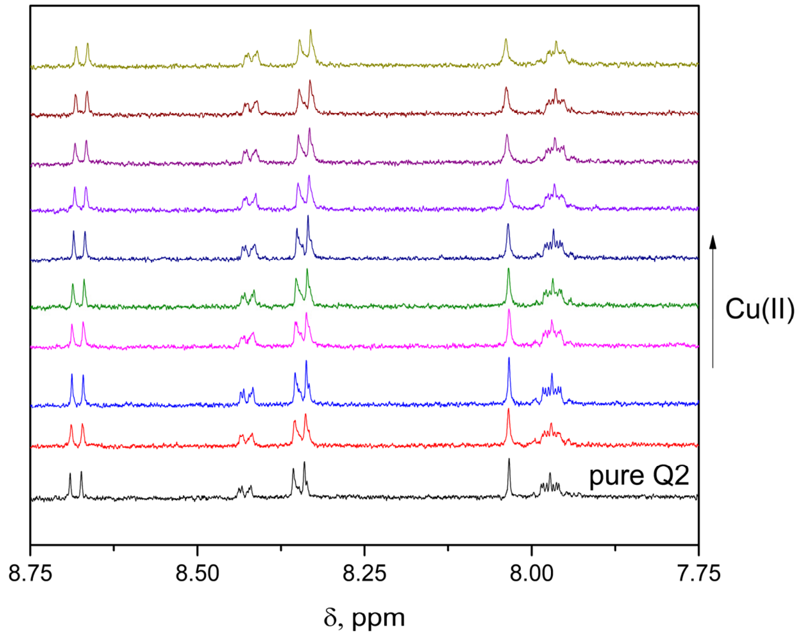

Figure S1. NMR studies of the interaction of Q2 with cupric ions in methanol. The ^{1}H NMR spectra of 0.5mM 4-hydroxynaphto [2,3-*h*]cinnoline-7,12-dione (Q2) in CD_3OD in the presence of $CuCl_2$ at concentrations in range 0.05-1mM. (The chemical shift (δ) is shown in the horizontal axis).

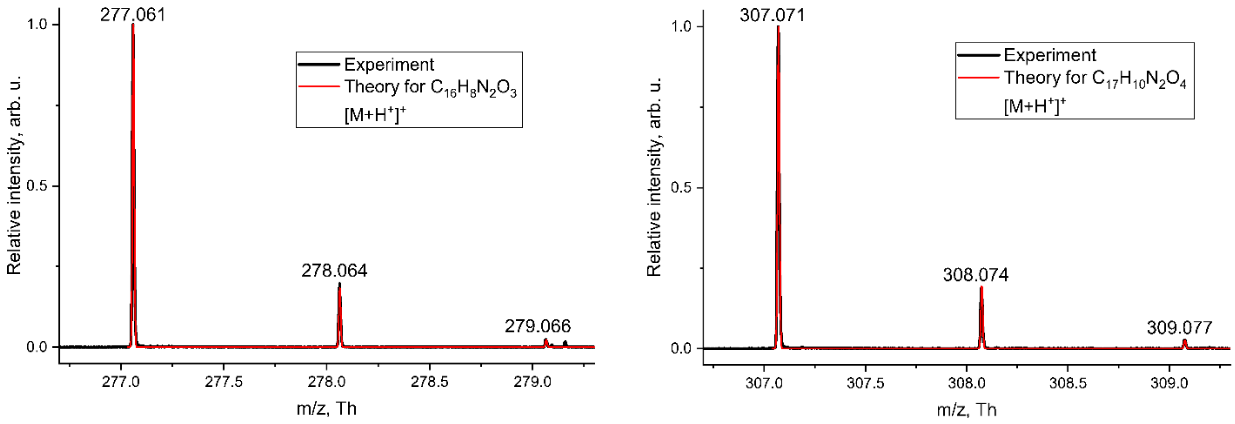

(**a**) (**b**)

Figure S2. HR-ESI-MS spectra of (**a**) Q2 and (**b**) Q3

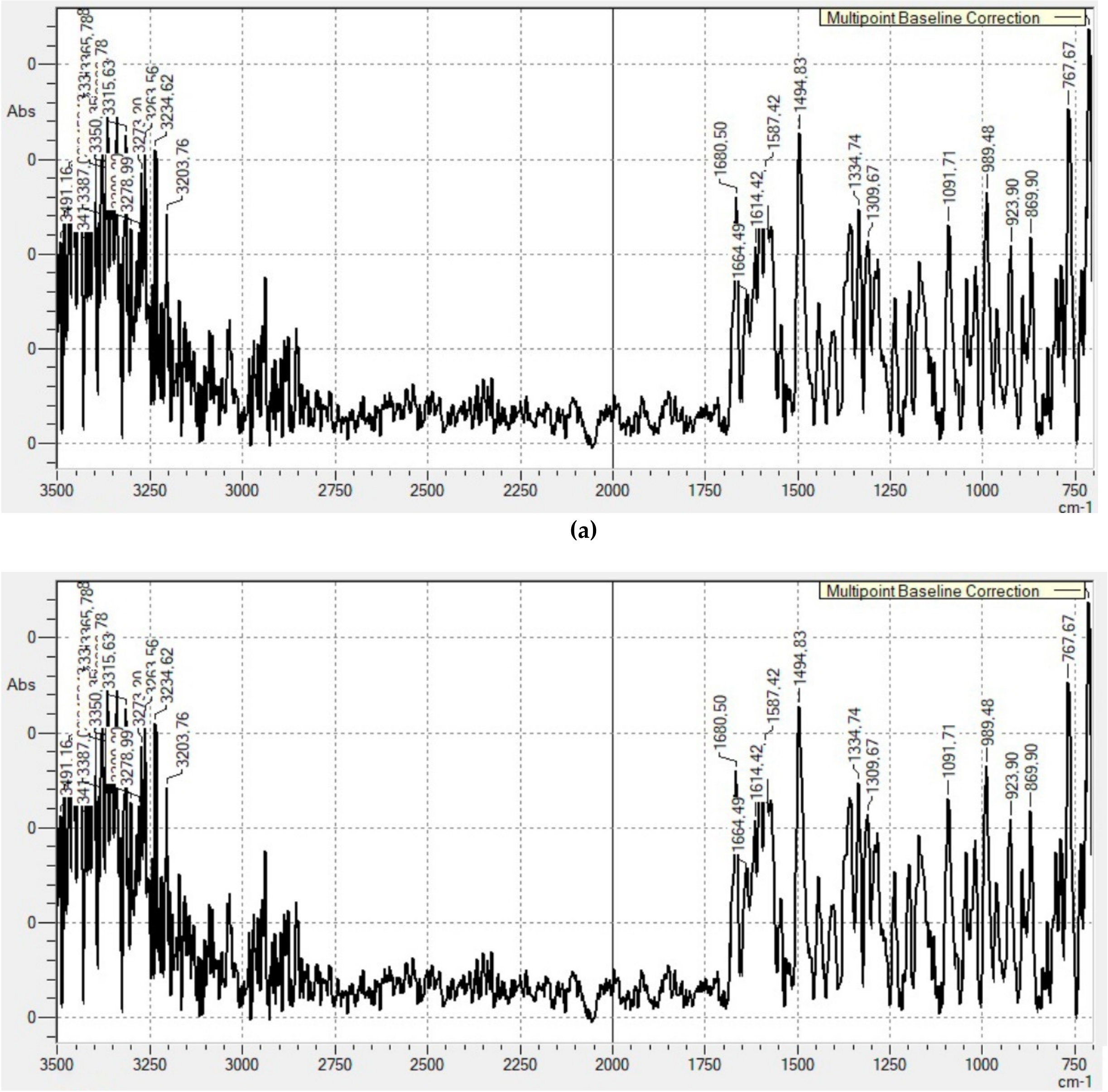

(a)

(b)

Figure S3. IR spectra of (**a**) Q2 and (**b**) Q3
